# Bibliometric analysis of literature on female genital mutilation: (1930 – 2015)

**DOI:** 10.1186/s12978-016-0243-8

**Published:** 2016-10-10

**Authors:** Waleed M. Sweileh

**Affiliations:** College of Medicine and Health Sciences, An-Najah National University, Nablus, Palestine State of Palestine

**Keywords:** Female genital mutilation, Africa, Middle East, Bibliometric analysis

## Abstract

**Background:**

Female genital mutilation/cutting (FGM/C) is a common harmful traditional practice in many communities in Africa and to a lesser extent in Middle East and other regions in the world. In order to better understand publishing on this topic, we conducted a bibliometric study on FGM/C. Bibliometric analyses can be used as an indicator of the extent of interaction of researchers, health authorities, and communities with a particular health issue.

**Methods:**

Scopus database was used to retrieve data on FGM/C. Keywords used were “female genital mutilation”, “female genital circumcision”, “female genital cutting” and “female circumcision”. Specifically, the number of publications, top productive countries and institutions, highly cited articles, citation analysis, co-authorships, international collaboration, role of African countries, top active authors, and journals involved in publishing articles on FGM/C were reviewed and analyzed. We indirectly assessed the impact of publications using total number of citations received, average number of citations per article, Hirsch-index, percentage of highly cited articles, and journal’s impact factor.

**Results:**

One thousand and thirty-five publications on FGM/C were retrieved. The h-index of retrieved articles was 37. A steep rise in number of publications was noticed in mid-1990s and again in 2012. More than half of retrieved articles were published from 2006 – 2015. A total of 65 countries contributed. The top ten productive countries included ones from Northern America, Europe and Africa. Nigeria and Egypt were the most active African countries in FGM/C publications. At least nine African academic institutions were actively involved on FGM/C publications. Articles on FGM/C that received the highest number of citations were those that focused on negative physical and psychosexual consequences of FGM/C. Journal topic areas were obstetrics/gynecology, public health, and psychological sociology. Collaboration between African and European countries on FGM/C research was evident.

**Conclusion:**

Bibliometric analysis reveals that research publications on FGM/C have been increasing since the l970s, with collaboration between African and Western countries, and articles are being published in higher impact journals, not only obstetrics, but also public health and social sciences. FGM/C research can be helpful to international health agencies and governments not only to document negative outcomes, but also to identify best practices, and to note gaps in implementation and practice.

## Plain English Summary

Female genital mutilation/cutting (FGM/C) is a common practice in many African and some Middle Eastern countries, as well as among some migrant groups in the United States of America (USA), Canada, Europe, and Australia. In addition to its potential serious acute and/or chronic health consequences, FGM/C is considered a violation of human rights. This study was carried out to assess worldwide research productivity on FGM/C, which can be used as an indicator of the extent of interaction of researchers, health authorities, and communities with a particular health issue. Our results showed that research productivity on FGM/C has increased markedly in the last two decades. African countries and institutions, particularly those in Nigeria and Egypt, had a noticeable number of publications. Many African countries contributed to FGM/C publications through international collaboration. Articles on FGM/C with the highest number of citations were those that focused on negative physical and mental health consequences of girls and women who were subjected to FGM/C. Many articles on FGM/C were published in gynecology/obstetrics journals, but also in public health and social sciences. Publications on FGM/C appeared in highly influential and prestigious journals emphasizing the international dimension and importance of this topic as a global public health issue.

## Background

Female genital mutilation/cutting (FGM/C) is a traditional practice that involves intentional removal of some or all of the external female genitalia for non-therapeutic purposes and with no health benefits for females [1]. There are different types of FGM/C depending on the extent of genital tissue removed. Types of FGM/C range from nicking the hood of the clitoris to clitoridoctomy to infibulation [2, 3]. The United Nations Children's Emergency Fund (UNICEF) indicated that although the prevalence of FGM/C has declined, the number of girls and women expected to undergo FGM/C will increase significantly if the practice continues at the same rates [4]. While the exact number of girls and women exposed to FGM/C is unknown, it is believed that at least 200 million women alive today have been subjected to FGM/C [4]. Of those 200 million girls and women, more than half were in Indonesia, Egypt and Ethiopia [4]. Physical and mental health consequences of FGM/C were investigated and researchers and clinicians concluded that FGM/C causes both acute and long-term harm [2, 5, 6]. Governmental and non-governmental organizations consider FGM/C a global public health concern and a health challenge, which could be eliminated by spread of knowledge, awareness, education, legislation, targeted programs, and research [7–9]. In line with international efforts aimed to completely eliminate this traditional harmful practice, research efforts on various aspects of FGM/C are needed.

Bibliometric analysis is a statistical tool used to assess the quantity and quality of publications, as well as the extent of success and achievements accomplished on a certain topic [10, 11]. Bibliometric analysis has been used in various medical disciplines in order to assess research trends and suggest future research ideas. Bibliometric analysis is important for young researchers to help them identify research leaders and issues. Furthermore, bibliometric analysis allows health policymakers to implement preventive measures, if, for example, bibliometric analysis indicates a rising number of articles on a certain issue or geographical location. For example, the dramatic increase in the number of publications on carabpenem resistance in certain types of bacteria was an alarming signal for those in the field of microbiology. Hundreds of bibliometric studies have been carried out and published on various medical subjects and on various challenging public health issues [12–15].

A literature search using Google Scholar, Pubmed, and Scopus for the period 1980 – 2016 found no bibliometric analysis or assessment of research output on FGM/C. The number of articles published on FGM/C from any particular country may be an indicator of how common this practice is in that country. On the other hand, the absence of publications on FGM/C from any particular country might suggest the absence of this practice. Therefore, bibliometric analysis of the source of research on FGM/C could be a tool for geographically mapping this traditional practice. We carried out this bibliometric study to assess the growth of publications, active countries and institutions, highly cited articles, citation analysis, co-authorships, international collaboration, role of African countries, top active authors, and journals involved in publishing articles on FGM/C to determine if it is indeed recognized as a growing public health problem.

## Methods

The methodology used in this study has been previously described in published bibliometric studies [16, 17]. Different electronic databases can be used to carry out bibliometric analysis. In this study, Scopus database was used because it has several advantages over other databases [18]. Scopus includes publications from all scientific, medical, and social disciplines in contrast to Pubmed, which includes only publications in medical and biomedical disciplines. Scopus allows data analysis such as citation analysis, country, author and organization/institution analysis. Such analysis is not available through Pubmed. Scopus is a larger database than Web of Science. Finally, Scopus provides more accurate data collection and analysis than Google scholar [19].

Keywords used to retrieve data included all terms used in literature to describe female genital mutilation. Such terms include “female genital cutting”, “female genital circumcision”, “female circumcision” and “female genital mutilation”. Search of these terms was confined to title search. Quotation marks were used to increase accuracy of search query. The time limit of the study was set from 1900 – 2015. Retrieved documents were limited to journal articles. Books, book chapters, and errata documents were all excluded. Retrieved data were analyzed for types of published documents, language, annual research productivity, top ten productive countries, institutions, citation analysis, collaboration analysis, top active authors and highly cited articles. Growth of publications was presented graphically using Statistical Package for Social Sciences software (SPSS). Indicators used to assess the scientific impact of retrieved data were the number of citation per article and the impact factor of the journal.

Scopus can be used to provide country analysis on any certain topic. An article in which all authors are from the same country is counted once for that country. However, for example, if a certain article has two authors from two different countries, then the article will be counted twice. Scopus allows researchers to count the number of articles produced by authors affiliated with the same country and articles produced by authors with different country affiliation. Single country publications (SCP) are articles in which all authors have the same country affiliation and such publications represent intra-country collaboration. Multiple country publications (MCP) are articles in which authors have different country affiliation and such publications represent inter-country collaboration. The standard competition ranking (SCR) was used to rank top ten productive countries, institutions, and authors. Whenever necessary, data pertaining to SCP and MCP were also presented.

Quality of publications is difficult to measure or assess directly. However, the impact of publications could be assessed indirectly using indicators such as total number of citations received, average number of citations per article, Hirsch-index (h-index), percentage of highly cited articles, and impact factor (IF) of journals publishing the documents of interest. H-index has been developed to assess productivity and citation impact of individual researchers [20]. However, the use of h-index has been extended to measure the productivity and citation impact of countries and academic institutions [20]. In this study, h-index for countries and institutions were obtained directly from Scopus database while IF was obtained from Journal Citation Report 2015 published by Thompson Reuters [21]. To visualize country collaboration and co-authorships, VOSviewer was used [22]. VOSviewer can present information either as density visualizations or network visualizations maps.

Ethical approval of this study was not required by IRB since no human subjects or data were involved. All data analysis was carried out on July 22nd, 2016 to avoid the dynamics of citations from one day to another.

## Results

### General information

A total of 1035 journal articles were retrieved. Of this number, 688 (66.47 %) were research articles. The remaining types of documents were review articles (131; 12.66 %), notes (74; 7.15 %), letters (62; 5.99 %), editorials (31; 3.0 %), short surveys (28; 2.71 %), conference papers (14; 1.35 %), and articles in press (7; 0.68). Retrieved articles were published in 15 different languages. English (931; 89.95 %) was the primary language, followed by French (31; 3.0 %) and German (22; 2.13 %). Retrieved articles had 7998 citations with an average of 7.73 citations per article. The h-index of retrieved articles was 37. The oldest publications on FGM/C were three letters appearing in *Lancet*, 1931 [23–25].

**Fig. 1:**
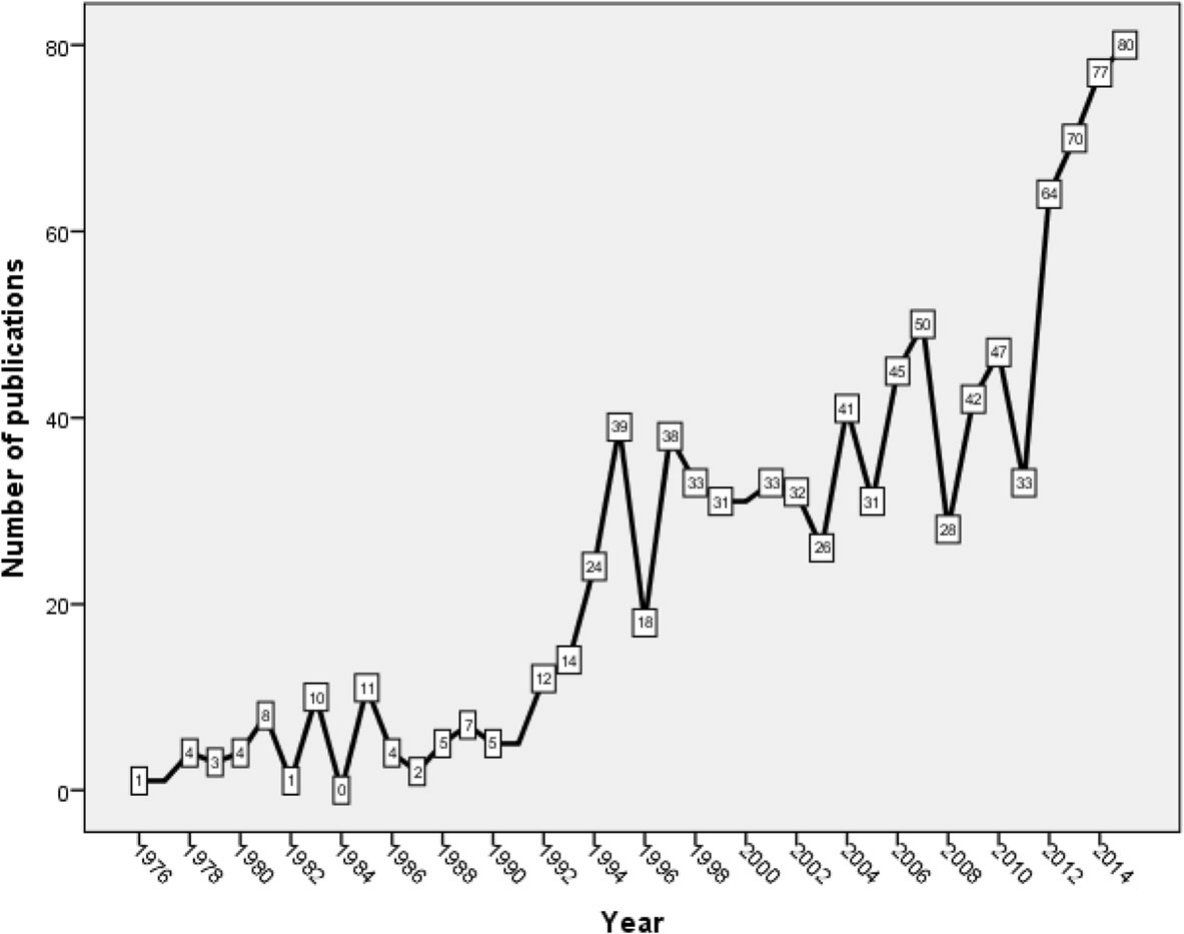
Growth of publications on FGM/C (1976 – 2015). Data from 1930 to 1975 were not shown in the Figure. FGM/C = female genital mutilation/cutting

Figure 1 shows the growth of publications on FGM/C from 1976 – 2015. The total number of publications in the 45 years from 1930 – 1975 was only 25, less than 1/year; therefore, these were not shown in Fig. 1. The number of publications on FGM/C showed an obvious increase in mid 1990s and again in 2012. The total number of publications produced from 2006 – 2015 was 536, approximately half (51.79 %) of retrieved articles. Table 1 shows annual research productivity and citation analysis of articles published during the last decade (2006 – 2015). There was a linear increase in cumulative number of citations with time. However, the number of citations per article per year showed an inverse linear relationship with time indicating that older articles were being continuously cited over time (Table 1)).

**Table 1:** Annual research output and citation analysis of FGM/C (2006 – 2015)

Year	Number of publications (%) *N* = 1035	TC	C/A	CC
2015	80 (7.73)	44	0.55	2493
2014	77 (7.44)	95	1.23	2449
2013	70 (6.75)	202	2.89	2354
2012	64 (6.18)	257	4.02	2152
2011	33 (3.19)	200	6.06	1895
2010	47 (4.54)	225	4.79	1695
2009	42 (4.06)	277	6.60	1470
2008	28 (2.71)	231	8.25	1193
2007	50 (4.83)	416	8.32	962
2006	45 (4.35)	546	12.13	546

*Abbreviation: FGM/C* female genital mutilation/cutting, *TC* total citations, *C/A* number of citations per article calculated by dividing the total number of citations retrieved for each year by the total number of publications in that year, *CC* cumulative citations calculated by adding up the number of citations for each year with the citations for all previous years

### Country analysis

A total of 65 countries contributed to the advancement of FGM/C research. Table 2 shows a list of all countries that contributed to FGM/C publications. The USA had the greatest share of publications, followed by the United Kingdom (UK), Nigeria, and Egypt. The list included 19 (29.23 %) African countries and three South American countries (Cuba, Colombia, and Venezuela). Among the world regions, Europe (343; 33.14 %) had the greatest share of publications followed by Northern America (188; 18.16 %), and Africa (173; 16.71 %). The total percentage of articles with country affiliation was 769 (74.30 %). The remaining articles (266; 25.70 %) had no country affiliation.

**Table 2:** List of countries and their share of publications on FGM/C

Country	Frequency *N* = 1035	Country	Frequency *N* = 1035	Country	Frequency *N* = 1035	Country	Frequency *N* = 1035
USA	162 (15.65)	South Africa	14 (1.35)	Cuba	2 (0.19)	Colombia	1 (0.10)
UK	116 (11.21)	Netherlands	12 (1.16)	Djibouti	2 (0.19)	Fiji	1 (0.10)
Nigeria	52 (5.02)	Ethiopia	10 (0.97)	India	2 (0.19)	Finland	1 (0.10)
Egypt	36 (3.48)	Kenya	9 (0.87)	Iran	2 (0.19)	Hungary	1 (0.10)
Australia	30 (2.90)	Denmark	8 (0.77)	Japan	2 (0.19)	Jordan	1 (0.10)
Sweden	30 (2.90)	Israel	8 (0.77)	Mali	2 (0.19)	Kiribati	1 (0.10)
Switzerland	30 (2.90)	Senegal	6 (0.58)	Oman	2 (0.19)	Kuwait	1 (0.10)
Italy	28 (2.71)	Burkina Faso	5 (0.48)	Qatar	2 (0.19)	Malawi	1 (0.10)
Germany	27 (2.61)	Gambia	4 (0.39)	Tanzania	2 (0.19)	New Zealand	1 (0.10)
Canada	26 (2.51)	Ghana	4 (0.39)	Uganda	2 (0.19)	Pakistan	1 (0.10)
France	21 (2.03)	Ireland	4 (0.39)	UAE	2 (0.19)	Poland	1 (0.10)
Spain	19 (1.84)	Malaysia	4 (0.39)	Austria	1 (0.10)	Portugal	1 (0.10)
Norway	17 (1.64)	Greece	3 (0.29)	Bangladesh	1 (0.10)	Sierra Leone	1 (0.10)
Sudan	17 (1.64)	Iraq	3 (0.29)	Bulgaria	1 (0.10)	Slovenia	1 (0.10)
Belgium	16 (1.64)	Tunisia	3 (0.29)	Cambodia	1 (0.10)	Swaziland	1 (0.10)
KSA	15 (1.45)	Botswana	2 (0.19)	China	1 (0.10)	Thailand	1 (0.10)
						Venezuela	1 (0.10)

*Abbreviations: FGM/C* female genital mutilation/cutting, *USA* United States of America, *UK* United Kingdome, *UAE* United Arab Emirates, *KSA* Kingdom of Saudi Arabia

**Fig. 2:**
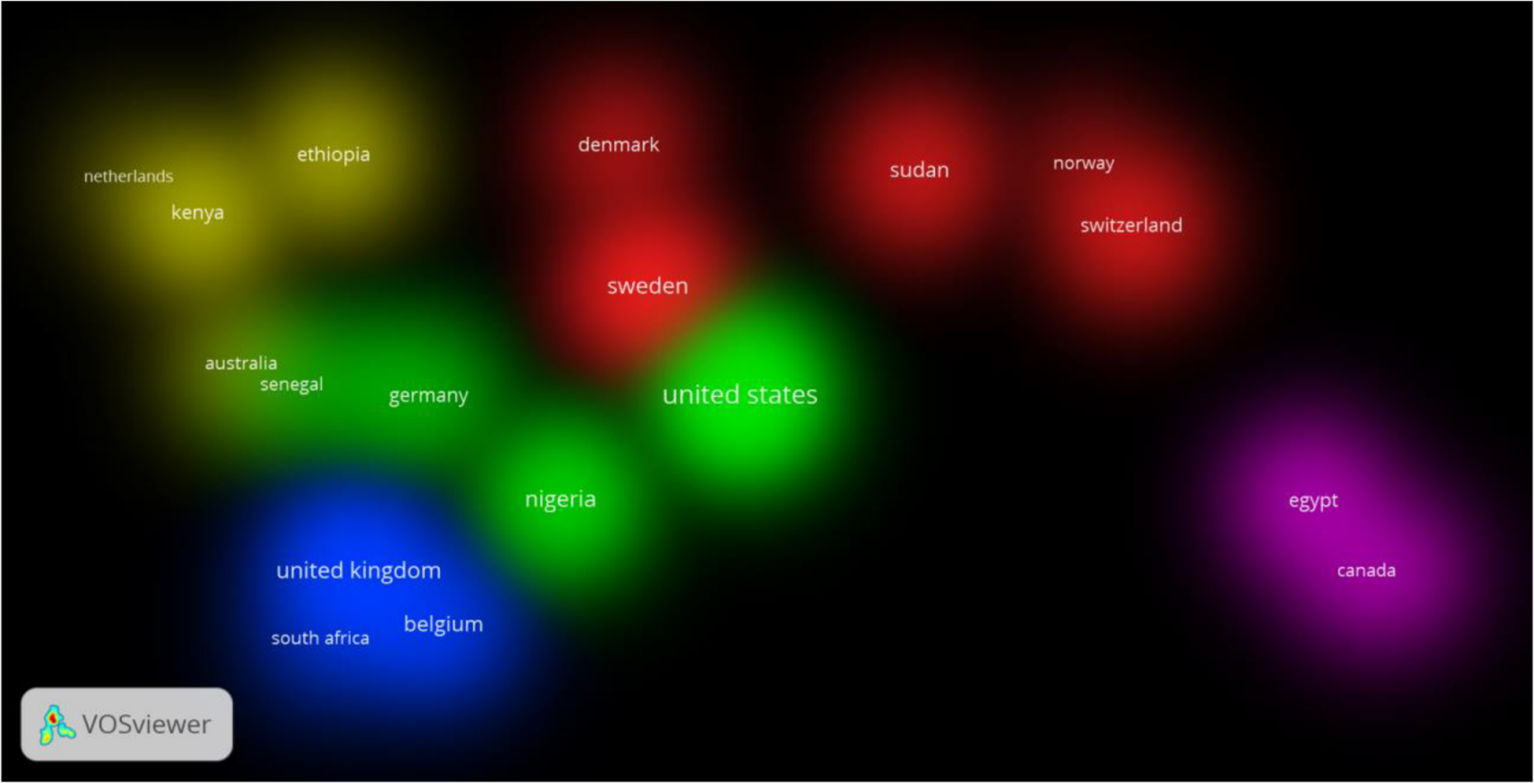
Density visualization of clusters of country co-authorships using VOSviewer for publications on FGM/C (1930 – 2015). Using a minimum threshold of 5 documents per country. The map included 21 countries

Top ten productive countries on FGM/C were shown in Table 3. Citation analysis and extent of international collaboration for each country in the top ten list are also shown. The total number of publications produced by top ten productive countries was 537 (51.88 %), which constituted more than half of worldwide productivity on FGM/C. The USA had the greatest share of publications, highest average number of citations per article, highest h-index value, and highest percentage of highly cited articles. Sweden had the greatest extent of international collaboration. More than one third (36.67 %) of articles published by Swedish researchers had co-authors from other countries. Visualization of international collaboration in FGM/C publications is shown in Fig. 2. Countries located within the same cluster have more research collaboration compared to countries outside the cluster. Furthermore, countries with higher number of co-authorships had a higher extent of international collaboration compared with countries having fewer co-authorships. Table 4 shows the number designated for each cluster in the visualization map and lists countries located within each cluster along with the number of co-authorships for each country.

**Table 3:** Citation analysis and research productivity of top 10 productive countries on FGM/C publications (1930 – 2015)

SCR^a^	Country	Number (%) *N* = 1035	TC	C/A	h-index	HCA (%)	NCC	SCP (%)	MCP (%)
1st	USA	162 (15.65)	2108	13.01	25	34 (30.0)	17	135 (83.33)	27 (16.67)
2nd	UK	116 (11.21)	861	7.42	16	9 (7.76)	12	101 (87.07)	15 (12.93)
3rd	Nigeria	52 (5.02)	438	8.42	12	7 (13.46)	7	41 (78.85)	11 (21.15)
4th	Egypt	36 (3.48)	495	13.75	13	6 (16.67)	5	30 (83.33)	6 (16.67)
5th	Australia	30 (2.90)	166	5.53	8	3 (10.00)	6	25 (83.33)	5 (16.67)
5th	Sweden	30 (2.90)	525	17.50	15	12 (40.00)	11	19 (63.33)	11 (36.67)
5th	Switzerland	30 (2.90)	289	9.63	10	7 (23.33)	5	24 (80.00)	6 (20.00)
8th	Italy	28 (2.71)	121	4.32	5	1 (3.57)	2	26 (92.86)	2 (7.14)
9th	Germany	27 (2.61)	237	8.78	7	4 (14.81)	5	22 (81.48)	5 (18.52)
10th	Canada	26 (2.51)	218	8.38	7	4 (15.38)	3	22 (84.62)	4 (15.38)

*Abbreviations: FGM/C* female genital mutilation/cutting, *TC* total citations, *C/A* number of citations per article calculated by dividing the total number of citations retrieved for each year by the total number of publications in that year, *CC* cumulative citations calculated by adding up the number of citations for each year with the citations for all previous years, *h-index* Hirsch index, *HCA* highly cited articles (those with ≥ 20 citations), *NCC* number of collaborating countries, *SCP* single country publication (intra-country collaboration), *MCP* multiple country publications (inter-country publications)

^a^SCR: Standard competition ranking. Equal countries were given the same ranking number, and then a gap is left in the ranking numbers

**Table 4:** List of countries, co-authorships, and colors of clusters present in visualization map in Fig. 3

Cluster 1 Country (Number of co-authorships)	Cluster 2 Country (Number of co-authorships)	Cluster 3 Country (Number of co-authorships)	Cluster 4 Country (Number of co-authorships)	Cluster 5 Country (Number of co-authorships)
^a^Red (6 items)	Green (5 items)	Blue (5 items)	Yellowish Green (4 items)	Purple (3 items)
Denmark (5)	Burkina Faso (4)	Belgium (10)	Australia (3)	Canada (4)
Italy (2)	Germany (6)	France (7)	Ethiopia (6)	Egypt (7)
Norway (2)	Nigeria (14)	South Africa (4)	Kenya (7)	KSA (3)
Sudan (8)	Senegal (4)	Spain (5)	Netherlands (1)	
Sweden (15)	USA (28)	UK (14)		
Switzerland (7)				

*Abbreviations*: *USA* United States of America, *UK* United Kingdom, *KSA* Kingdom of Saudi Arabia

^a^refers to the colors shown in Fig. 3

The share of specific African countries to FGM/C publications was investigated. Table 5 shows that 14 African countries from the 27 African countries listed by UNICEF as having high rates of FGM/C had contributed to FGM/C publications. The number of publications authored or co-authored by authors from the 14 African countries was 151, representing 14.89 % of worldwide publications on FGM/C. Approximately 40 % of articles published by these specific African countries were produced through international collaboration. Nigeria and Egypt were the most productive African countries.

**Table 5:** Contribution of specific African countries to FGM/C publications (1930 – 2015)

No	Country^a^	Number of publications	SCP	MCP
1	Burkina Faso	5	1 (20)	4 (80.0)
2	Djibouti	2	0 (0.0)	2 (100.0)
3	Egypt	36	30 (83.33)	6 (16.77)
4	Ethiopia	10	4 (40.0)	6 (60.0)
5	Gambia	4	0 (0.0)	4 (100)
6	Ghana	4	1 (25.0)	3 (75.0)
7	Kenya	9	3 (33.33)	6 (66.77)
8	Mali	2	0 (0.0)	2 (100.0)
9	Nigeria	52	41 (78.85)	11 (21.15)
10	Senegal	6	2 (33.33)	4 (66.77)
11	Sierra Leone	1	0 (0.0)	1 (100)
12	Sudan	17	9 (52.94)	8 (47.06)
13	Uganda	2	0 (0.0)	2 (100.0)
14	United Republic of Tanzania	1	0 (0.0)	1 (100.0)
	Total	151	91 (60.26)	60 (39.74)

*FGM/C* female genital mutilation/cutting, *SCP* single country publication, *MCP* multiple country publications

^a^Countries were selected based on the UNICEF list of African countries in which FGM/C is a common practice. Only African countries were studied. The following African countries have zero contribution to FGM/C publications and were not listed in the table: Benin, Cameroon, Central African Republic, Chad, Côte d'Ivoire, Eritrea, Guinea, Guinea-Bissau, Liberia, Mauritania, Niger, Somalia, and Togo

### Institutions/Organizations

Institutions and organizations with at least five publications on FGM/C are shown in Table 6. The list included nine African academic institutions. The top ten active institutions/ organizations were shown in Table 7. The most active institution was *Karolinska Institutet* in Sweden. Publications of the University of Washington, USA had the highest average number of citations per article (22.56) and the highest percentage of publications that were highly cited (33.33 %). King Saud University (KSA), Kingdom of Saudi Arabia was among the top ten productive institutions. Other institutions in the top ten productive list included those in the UK and the USA. The WHO was also among the top ten productive institutions/organizations.

**Table 6:**
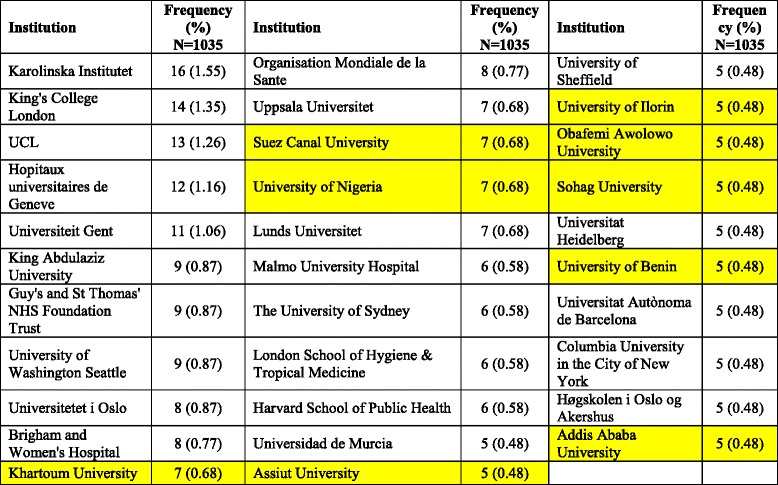
List of active institutions with at least 5 published articles on FGM/C (1930 – 2015)

*FGM/C* female genital mutilation/cutting

Yellow highlight represents institution in Africa

**Table 7:** Productivity and citation analysis of top ten productive institutions/organizations on FGM/C (1930 – 2015)

SCR^a^	Institution^b^ (country affiliation)	Frequency (%) *N* = 1035	TC	C/A	h-index	HCA
1st	*Karolinska Institutet* (Sweden)	16 (1.55)	226	14.13	8	4 (25.00)
2nd	*King's College London* (UK)	14 (1.35)	96	6.86	5	1 (7.14)
3rd	*UCL* (UK)	13 (1.26)	63	4.85	5	0 (0.0)
4th	*Hopitaux universitaires de Geneve* (Switzerland)	12 (1.16)	61	5.08	3	1 (8.33)
5th	*Universiteit Gent* (Belgium)	11 (1.06)	98	8.91	6	1 (9.09)
6th	*King Abdulaziz University* (KSA)	9 (0.87)	117	13.00	5	2 (22.22)
6th	*Guy's and St Thomas' NHS Foundation Trust* (UK)	9 (0.87)	82	9.11	5	1 (11.11)
6th	*University of Washington Seattle* (USA)	9 (0.87)	203	22.56	7	3 (33.33)
9th	*Universitetet i Oslo* (Norway)	8 (0.77)	126	15.75	6	2 (25.0)
9th	*Brigham and Women’s Hospital* (USA)	8 (0.77)	113	14.13	5	2 (25.0)
9th	*Organisation Mondiale de la Sante* (WHO)	8 (0.77)	79	9.88	4	2 (25.0)

FGM/C: female genital mutilation/cutting

Abbreviations: SCR: Standard competition ranking: TC: total citations. C/A: number of citations per article calculated by dividing the total number of citations retrieved for each year by the total number of publications in that year. h-index: Hirsch index. HCA: highly cited articles (those with ≥ 20 citations).

UCL: University College London

^a^SCR: Standard competition ranking. Equal countries were given the same ranking number, and then a gap is left in the ranking numbers.

^b^Names of institutions were written the same way they appeared in Scopus.

### Journals

Journals that had published at least 5 articles on FGM/C were shown in Table 8. Retrieved articles were mainly published in general medical, obstetrics/ gynecology, and in public health journals. Journals that had the largest share of publications on FGM/C were *BMJ (Clinical Research Ed.)*, *Lancet* and *International Journal of Gynecology and Obstetrics.* Table 9 shows the impact factor and citation analysis for top ten productive journals in the field of FMG/C. The first three top productive journals are well-known journals with high IF. The total number of articles published by the top ten productive journals was 201 (20 %). The total IF of these articles was 2675.45 giving an average of 13.31 per article.

**Table 8:** List of journals that published at least 5 articles on FGM/C (1930 – 2015)

Journal^a^	Frequency (%) *N* = 1035	Journal	Frequency (%) *N* = 1035		Frequency (%) *N* = 1035
BMJ g Clinical Research Ed	39 (3.77)	Acta Obstetricia Et Gynecologica Scandinavica	8 (0.77)	Tropical Medicine and International Health	6 (0.58)
Lancet	39 (3.77)	Culture Health and Sexuality	8 (0.77)	Australian and New Zealand Journal of Obstetrics and Gynaecology	5 (0.48)
International Journal of Gynecology and Obstetrics	33 (3.19)	East African Medical Journal	8 (0.77)	BMJ	5 (0.48)
Journal of Obstetrics and Gynaecology	18 (1.74)	Lakartidningen	8 (0.77)	Bulletin of the World Health Organization	5 (0.48)
African Journal of Reproductive Health	14 (1.35)	Studies in Family Planning	8 (0.77)	Genus	5 (0.58)
Journal of Sexual Medicine	13 (1.26)	British Journal of Midwifery	7 (0.68)	Journal De Gynecologie Obstetrique Et Biologie De La Reproduction	5 (0.48)
BMC Public Health	12 (1.16)	CMAJ	7 (0.68)	Journal of Sex and Marital Therapy	5 (0.48)
BMJ Online	12 (1.16)	European Journal of Contraception and Reproductive Health Care	7 (0.68)	Medical Journal of Australia	5 (0.48)
African Journal 0f Urology	11 (1.06)	Health Care for Women International	7 (0.68)	Nigerian Journal of Medicine Journal of the National Association of Resident Doctors of Nigeria	5 (0.48)
Tropical Doctor	10 (0.97)	American Journal of Obstetrics and Gynecology	6 (0.58)	Nursing Standard Royal College of Nursing Great Britain 1987	5 (0.48)
BJOG an International Journal of Obstetrics and Gynaecology	9 (0.87)	British Journal of Obstetrics and Gynaecology	6 (0.58)	Obstetrical and Gynecological Survey	5 (0.48)
British Medical Journal	9 (0.87)	Medical Anthropology Quarterly	6 (0.58)	Practising Midwife	5 (0.48)
International Journal of Women’s Health	9 (0.87)	Midwifery	6 (0.58)	Sexologies	5 (0.48)
Obstetrics and Gynecology	9 (0.87)	Nederlands Tijdschrift Voor Geneeskunde	6 (0.58)	Women and Health	5 (0.48)
Reproductive Health Matters	9 (0.87)	New England Journal of Medicine	6 (0.58)		
Social Science and Medicine	9 (0.87)				

FGM/C: female genital mutilation/cutting.

^a^Journal names were shown in the table in the same way they appeared in Scopus

**Table 9:** Citation analysis and impact factor of top ten productive journals on FGM/C publications (1930 – 2015)

SCR^a^	Journal	Frequency (%) *N* = 1035	TC	C/A	h-index	IF	Total IF^b^
1st	*BMJ Clinical Research Ed*	39 (3.77)	85	2.179	5	19.967	778.71
1st	*Lancet*	39 (3.77)	432	11.077	9	44.002	1716.08
3rd	*International Journal of Gynecology and Obstetrics*	33 (3.19)	493	14.939	14	1.674	55.24
4th	*Journal of Obstetrics and Gynaecology*	18 (1.74)	133	7.389	7	0.611	11.00
5th	*African Journal of Reproductive Health*	14 (1.35)	70	5.000	5	0.91	12.74
6th	*Journal of Sexual Medicine*	13 (1.26)	111	8.538	6	3.151	40.96
7th	*BMC Public Health*	12 (1.16)	61	5.083	4	2.209	26.51
7th	*BMJ Online*	12 (1.16)	22	1.833	3	2.562	30.74
9th	*African Journal of Urology*	11 (1.06)	21	1.909	2	N/A	0.00
10th	*Tropical Doctor*	10 (0.97)	121	12.100	5	0.347	3.47
	Total	201 (19.42)					2675.46

*FGM/C* female genital mutilation/cutting

*Abbreviations: SCR s*tandard competition ranking, *TC* total number of citations, *C/A* number of citations per article calculated by dividing the total number of citations retrieved for each year by the total number of publications in that year, *h-index* Hirsch index, *HCA* highly cited articles (those with ≥ 20 citations), *IF*impact factor

^a^ *SCR,* Standard competition ranking, Equal countries were given the same ranking number, and then a gap is left in the ranking numbers

^b^Total impact factor was obtained by multiplying the number of articles published by a certain journal with the IF of that journal

### Top cited articles

Top 10 cited articles on FGM/C were shown in Table 10. The article which received the highest number of citations was “*Female genital mutilation and obstetric outcome: WHO collaborative prospective study in six African countries”* published in 2006 in *Lancet*. The article received a total of 221 citations. Seven of the top 10 cited articles were original articles and three were review articles. Most articles in the top cited list discussed reproductive and psychological/emotional/sexual consequences of FGM/C on girls and women. One article was on epidemiology, one was about public health aspect of FGM/C and one was about medicalization of FGM/C.

**Table 10:** List of highly cited articles on FGM/C (1930 – 2015)

SCR^a^	Authors	Title	Source title	Number of citations
1st	Banks, E. [49]	“Female genital mutilation and obstetric outcome: WHO collaborative prospective study in six African countries”	*Lancet*	221
2nd	Toubia N. [50]	“Female circumcision as a public health issue”	*New England Journal of Medicine*	180
3rd	Morison L. [51]	“The long-term reproductive health consequences of female genital cutting in rural Gambia: A community-based survey”	*Tropical Medicine and International Health*	87
4th	Dirie M.A., Lindmark G. [52]	“The risk of medical complications after female circumcision”	*East African Medical Journal*	87
5th	Shell-Duncan B. [53]	“The medicalization of female 'circumcision': Harm reduction or promotion of a dangerous practice?”	*Social Science and Medicine*	85
6th	Obermeyer C.M.[54]	“The consequences of female circumcision for health and sexuality: An update on the evidence”	*Culture, Health and Sexuality*	76
7th	Chalmers B., Hashi K.O. [55]	“432 Somali women's birth experiences in Canada after earlier female genital mutilation”	*Birth*	74
8th	De Silva S. [56]	“Obstetric sequelae of female circumcision”	*European Journal of Obstetrics and Gynecology and Reproductive Biology*	73
9th	Jones H. [57]	“Female genital cutting practices in Burkina Faso and Mali and their negative health outcomes”	*Studies in Family Planning*	67
10th	Behrendt A., Moritz S. [58]	“Posttraumatic stress disorder and memory problems after female genital mutilation”	*American Journal of Psychiatry*	64

*FGM/C* female genital mutilation/cutting

^a^ *SCR* standard competition ranking. Equal countries were given the same ranking number, and then a gap is left in the ranking numbers

### Authors

**Fig. 3:**
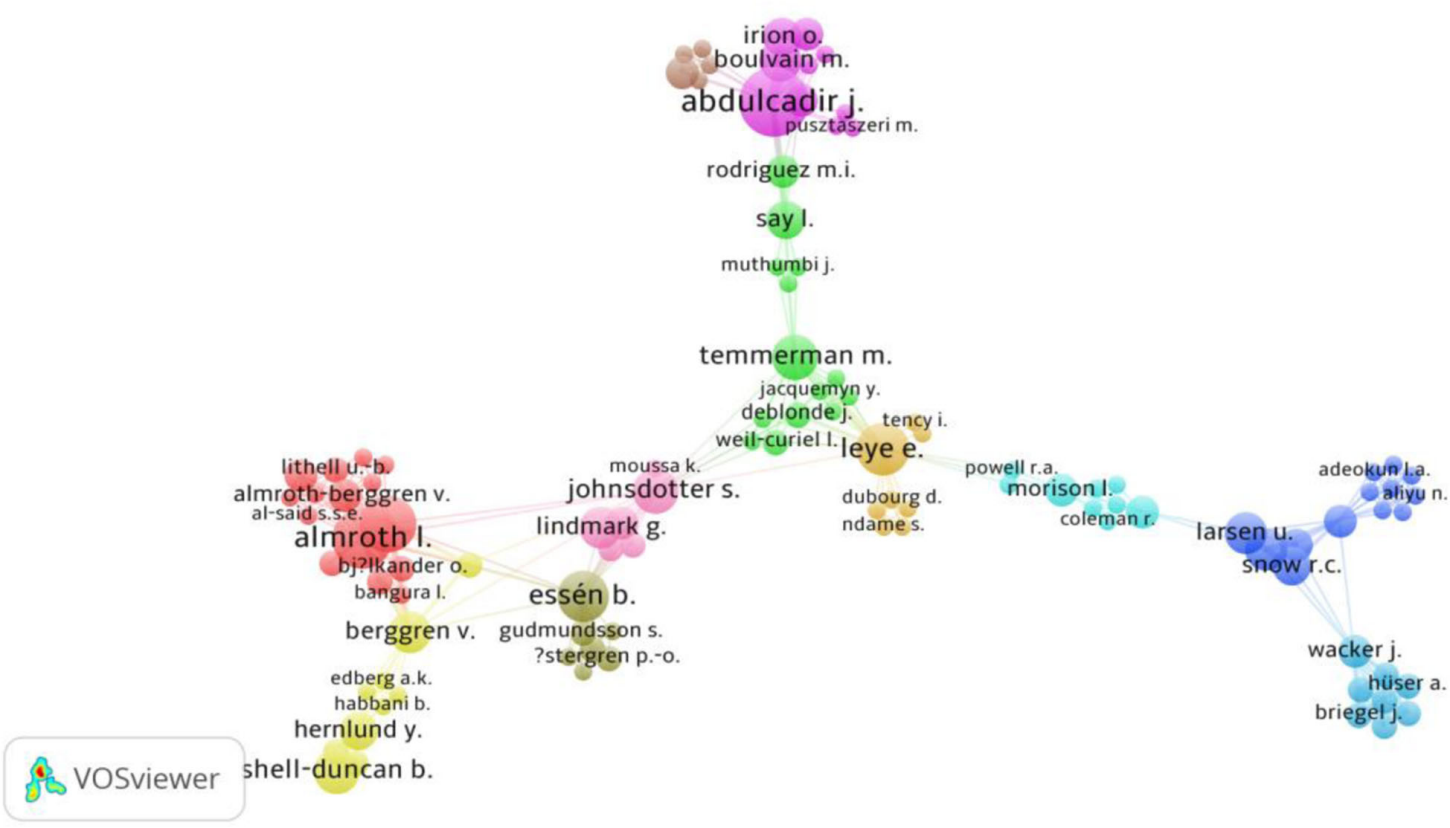
Network visualization map for author/co-authorship on FGM/C publication (1930 – 2015). Map included 125 authors

At least 1726 authors participated in publication of retrieved articles. Therefore, the minimum average number of authors per article was 1.7. Of course it would be impossible to list all authors of retrieved articles in this study. However, we used the VOSviewer to present authors with highest number of links with other authors, i.e., have high number of co-authorships. VOSviewer, might not show authors with low or no links, even if they have large number of published documents and therefore one should be cautious when interpreting VOSviewer maps. A total of 500 authors with the highest number of links were selected and analyzed by VOSviewer. Of those 500, the largest set of 125 authors were shown in a network visualization map. The map consisted of 11 clusters. In the map, authors with the highest number of links were shown with larger font size and larger circle size (Fig. 3). Professor Abdulcadir, J at *Universite de Geneve Faculte de Medecine, Faculty of Medicine, Geneve, Switzerland* was the most productive author with 13 publications and 31 co-authorships. Table 11 lists top ten active authors along with the number of documents they had published, number of co-authorships, and location of each author in the visualization map.

**Table 11:** Top 10 active authors and their location in network visualization map (Fig. 3)

SCR^a^	Author	Number of documents as retrieved from VOSviewer	% *N* = 1035	Number of Co-authorships based on 126 authors selected by VOSviewer	Cluster^b^
1st	Abdulcadir, J.	13	1.256	31	5
2nd	Rouzi, A.A.	10	0.966	0	Not shown
2nd	Almroth, L.	10	0.676	33	1
4th	Nour, N.M.	9	0.870	0	Not shown
4th	Creighton, S.M.^c^	9^c^	0.870	9^c^	Not shown
4th	Bergström, S.	9	0.580	31	1
4th	Bergström, S.	9	0.580	31	1
8th	Dyer, C.	8	0.773	0	Not shown
8th	Leye, E.	8	0.773	24	8
10th	Johnsdotter, S.	6	0.580	19	10
10th	Momoh, C.	6	0.580	4	Not shown
10th	Rymer, J.	6	0.580	2	Not Shown
10th	Shell-Duncan, B.	6	0.580	7	4

*FGM/C* female genital mutilation/ cutting

^a^ *SCR* Standard competition ranking. Equal countries were given the same ranking number, and then a gap is left in the ranking numbers

^b^represents the number of cluster in network visualization map created by VOSviewer (Fig. 3)

^c^author might have two profiles in Scopus. The data in table represent the sum of the two profiles

## Discussion

The aim of this study was to give a bibliometric overview of publications on FGM/C and how research on FGM/C had evolved in the past decades. Publication activity on FGM/C could be found starting in 1931 [23–25]. However, the number of publications on FGM/C remained extremely low until the late 1970s [23–26]. The WHO held its first international conference on FGM/C in Sudan in 1979 and took a firm stand against this practice by issuing a joint statement with UNICEF and the United Nations Population Fund (UNFPA) against the practice of FGM/C [27]. Since that time, international opposition to FGM/C has increased and great efforts have been made to stop this practice. Campaigns against FGM/C started in Africa and focused primarily on the negative health consequences of FGM/C [28–32]. These campaigns encouraged researchers and clinicians to discuss and publish on medical, social, psychiatric, religious, and legislative aspects of FMG/C. International efforts to counteract FGM/C made substantial progress at international and national levels. Both social and political momentum against FGM/C successfully led to adoption of anti-FGM/C legislation in at least 26 African countries and 33 Western countries.

Starting from 2003, the United Nations began sponsoring an “International Day of Zero Tolerance to Female Genital Mutilation,” which is held every 6 February [33]. Several joint reports and statements by international agencies contributed to abandonment of FGM/C [4]. Such joint reports include: (1) “Eliminating female genital mutilation: an interagency statement” issued in 2008 by WHO and nine other United Nations agencies; (2) "Global strategy to stop health care providers from performing female genital mutilation" issued in 2010; (3) UN General Assembly resolution 67/147 issued in late 2012; (4) an updated report on FGM/C issued by UNICEF in 2016; and (5) “Guidelines on the Management of Health Complications from Female Genital Mutilation” issued by WHO in 2016. During the 1990s, at least five human rights conventions were issued to protect women’s rights for their bodily integrity [34]. The 1990s also witnessed several international conferences that issued resolutions and plans to eradicate FGM/C. Such conferences include the 1995 International Conference on Population and Development and the 1995 Fourth World Conference on Women. Opposition to FGM/C was also endorsed by several African non-governmental organizations (NGOs) [34], which are heavily involved in educational campaigns against FGM/C. International opposition, educational campaigns, joint statements, conferences, and the social work of African NGOs may have contributed to the rise in the number of publications on FGM/C seen in this study.

International efforts and active campaigns against FGM/C led to a decline in the prevalence of FGM/C in Africa and other parts of the world [35, 36]. However, the number of girls and women who are at risk of undergoing FGM/C did not change and might actually increase in the future due to an increase in population size. The elimination of FGM/C requires not only spread of awareness among people but also strong community involvement.

The continuing practice of FGM/C among certain immigrant communities in Western countries has been reported [37–44]. The US Centers for Disease Control and Prevention (CDC) estimated that more than half a million women and girls in the USA had been subjected to FGM/C. This number represents a threefold increase from previous estimations made in 1990 [45]. The CDC attributed this change to increased immigration from countries in which FGM/C is a common practice. Healthcare systems and healthcare providers in many Western countries have had to face the health aspects and legislative aspects of FGM/C among migrant communities. Participation of researchers and clinicians in the West in the medical, social, and legislative aspects of FGM/C may have contributed to the increase in the number of publications on FGM/C.

As expected, analysis of highly cited articles showed that the majority were about the health consequences, both physical and mental, on girls and women with FGM/C. However, authors also published on different social, religious, and anthropological aspects of FGM/C [46–48]. The fact that the h-index of retrieved articles was 37, suggests that this topic draws some global attention; however, the h-index of FGM/C articles is lower than that reported for other reproductive health issues such as the use of emergency contraceptive pills [12].

International collaboration on FGM/C publication was shown in VOSviewer maps, which indicated the presence of strong collaboration between African and Western countries. Such international collaboration may aid African countries to recruit international agencies and funded programs to help eliminate the practice. Most countries with high prevalence of FGM/C are lower-resourced and international collaboration in creating public awareness about FGM/C is needed.

To the best of the author’s knowledge, this is the first bibliometric study on FGM/C. However, the author acknowledges the presence of some limitations in this study. The fact that one fourth of retrieved articles had no country affiliation made analysis pertaining to country profile relatively inaccurate and might under-estimate the actual number of publication for each country. Lack of country affiliation in some retrieved articles might be due to a delay in Scopus updating the system or due to publications authored by international organizations such the World Health Organization (WHO) or UNICEF. There are local and regional journals in Africa and Middle East that are not indexed in Scopus. Self-citation is common in publications and needs to be taken into consideration when interpreting results of citation analysis for countries and institutions. Finally, results pertaining to active authors might not be 100 % accurate because some authors use different name spelling, middle initials, etc. and therefore results will be affected.

## Conclusion

Assessing research productivity and scientific impact of FGM/C publications can be considered a new addition to FGM/C literature. This study shows that literature on FGM/C is increasing, and being published in some high impact journals, indicating recognition of FGM/C as a global public health concern. International collaboration had a positive impact on research productivity from lower resourced African countries, where the majority of FGM/C occurs. Further research from different countries, cultures, organizations, and individuals focusing on knowledge, awareness, legalization, opinions of lay and religious people, particularly including women rights activists, and women with FGM/C, are needed to widen and enrich the literature on FGM/C. This bibliometric analysis may be a first step to tracking the growth and quality of FGM/C research and literature.

## Abbreviations

FGM/C: Female genital mutilation/ cutting
UNICEF: United Nations Children’s Emergency Fund
WHO: World Health Organization
